# Radiation-Induced Testicular Injury and Its Amelioration by *Tinospora cordifolia* (An Indian Medicinal Plant) Extract

**DOI:** 10.1155/2011/643847

**Published:** 2011-02-16

**Authors:** Priyanka Sharma, Jyoti Parmar, Priyanka Sharma, Preeti Verma, P. K. Goyal

**Affiliations:** Radiation & Cancer Biology Laboratory, Department of Zoology, University of Rajasthan, Jaipur 302 004, India

## Abstract

The primary objective of this investigation is to determine the deleterious effects of sub lethal gamma radiation on testes and their possible inhibition by *Tinospora cordifolia* extract (TCE). For this purpose, one group of male Swiss albino mice was exposed to 7.5 Gy gamma radiation to serve as the irradiated control, while the other group received TCE (75 mg/kg b. wt./day) orally for 5 consecutive days half an hr before irradiation to serve as experimental. Exposure of animals to 7.5 Gy gamma radiation resulted into significant decrease in body weight, tissue weight, testes- body weight ratio and tubular diameter up to 15 days of irradiation. Cent percent mortality was recorded by day 17th in irradiated control, whereas all animals survived in experimental group. TCE pretreatment rendered significant increase in body weight, tissue weight, testes- body weight ratio and tubular diameter at various intervals as compared to irradiated group. Radiation induced histological lesions in testicular architecture were observed more severe in irradiated control then the experimental. TCE administration before irradiation significantly ameliorated radiation induced elevation in lipid peroxidation and decline in glutathione concentration in testes. These observations indicate the radio- protective potential of *Tinospora cordifolia* root extract in testicular constituents against gamma irradiation in mice.

## 1. Introduction

The threat of accidental or hostile exposure to radiation is of great concern. Ionizing radiation inflicts its adverse effects through the generation of oxidative stress that unleash large-scale destruction or damage of various biomolecules. Disorders of reproduction and hazards to reproductive health have become prominent issues in recent past after several reports of adverse effects of ionizing radiation on reproductive functions [1].

Oxidative stress is a major factor in the etiology of male infertility. Since the cells of spermatogenic lineage are especially vulnerable to radiation-induced reactive oxygen species (ROS) because they are constantly under mitosis or meiosis. This makes testes a highly radiosensitive organ with wide range of radiosensitive germ cells [2]. Under normal conditions, the testis is afforded with antioxidant protection as an elaborate array of antioxidant enzymes, free radical scavengers, and low oxygen tension in order to support the dual action of this organ as germ cells spermatogenic [3] as well as Leydig cells steroidogenic function [4]. However, a wide variety of endogenous and exogenous factors are known that are capable to perturb these defenses and compromise male fertility by generating free radicals in testes [5, 6]. 

With the increasing incidences of radiation-induced reproductive disorders, there is an urgent need to identify a radio-protector that is effective in multidirectional manner and can supplement the tissue's own antioxidant strategies to rescue the testes from the deleterious consequences of ROS attack. Research in the development of radio-protectors worldwide has focused on screening a variety of chemical and biological compounds in both *in vitro *and *in vivo *models [7–11]. The application of these molecular or synthetic drugs is limited in diverse fields of radiation owing their unacceptable level of the toxicity to one or more vital body systems at the effective concentration. However, decades of research failed to deliver a radioprotective drug which meets all the prerequisites of an ideal radio-protector. In view of this, the search for less or nontoxic and more potent radio-protector drug is continued. Herbal medicine offers an ultimate alternative to their synthetic counterparts [12].

Several phytoceuticals and plant extracts with innumerable pharmacological properties in recent past years have been reported to act as good radio-protector due to the ability of scavenging the free radicals and modulating antioxidant defense system of the body by up/downregulation of the antioxidant gene expression [13]. Medicines derived from plants have played a pivotal role in health care of ancient and modern cultures. Current estimate indicates that about 80% of people in developing countries still rely on traditional medicinal plant-based remedies to meet their primary healthcare [14]. Some medicinal plants have been trialed for their antiradiation property and many of them are found to be useful [15, 16].

*Tinospora cordifolia *(Family: Menispermaceae) has been appreciated as an important drug of the Indian system of medicine and used in Ayurvedic preparations for the treatment of various ailments. It possesses antistress, antidiabetic, antiulcer, anti-oxidative, radioprotective, hepatoprotective, immunomodulator, and learning and memory enhancing properties [17–19]. A variety of bioactive constituents, with anti-oxidative and free radical scavenging properties, have been isolated from *T. cordifolia *[20–22].

Looking towards the above pharmacological and therapeutic properties, present study has been undertaken to find out the possible radioprotective potential of the *Tinospora cordifolia *extract (TCE) against radiation-induced testicular injury in Swiss albino mice.

## 2. Materials and Methods

### 2.1. Animal Care and Handling

The animal care and handling were performed according to the guidelines set by the WHO (World Health Organization, Geneva, Switzerland) and the INSA (Indian National Science Academy, New Delhi, India). Swiss albino mice, 6–8 weeks old weighing 22 ± 2 gm from an inbred colony, were used in the present study. They were maintained under controlled conditions of temperature and light (14 and 10 hrs of light and dark, resp.). The animals were provided with standard mice feed (procured from Ashirwad Industries, Chandigarh, India) and water *ad libitum*. Tetracycline water was also given once a fortnight as a preventive measure against infection. Four to six animals were housed in a polypropylene cage containing paddy husk (procured locally) as a bedding throughout the experiment. The Institutional Animal Ethical Committee approved the study.

### 2.2. Source of Irradiation

Animals were irradiated by a Co^60^ source in the cobalt therapy unit at Cancer Treatment Center, Department of Radiotherapy, SMS Medical College and Hospital, Jaipur, India. Unanaesthestized mice were restrained in well-ventilated boxes and exposed whole body to gamma radiation (7.5 Gy) at the dose rate of 221 c Gy/min from the source to surface distance (SSD), that is, 80 cm.

### 2.3. Preparation of the Plant Extract

*Tinospora cordifolia *was identified by a competent Botanist in Herbarium of Botany Department, University of Rajasthan, Jaipur (RUBL No. 20132). Root of the *Tinospora cordifolia* was collected, cleaned, shade dried, powdered, and extracted. The extract was prepared by refluxing with double-distilled water (DDW) for 36 (12 × 3) hours. The cooled liquid extract was concentrated by evaporating its liquid contents to render it in powder form. An approximate yield of 22% extract was obtained. The extract was redissolved in DDW just before oral administration in mice. Henceforth in this paper, the extract of *Tinospora cordifolia *root extract will be called TCE.

### 2.4. Dose Selection of TCE

Dose selection of *Tinospora cordifolia *was done in our previous study on the basis of drug tolerance survival experiment [23].

### 2.5. Experimental Design

To evaluate the adverse effects of gamma rays and the possible radioprotective efficacy of TCE extract, male Swiss albino mice were selected from an inbreed colony and randomly divided into following groups.

**Group I (normal/sham-irradiated)**

Mice (*n* = 8) of this group were given double distilled water (DDW) dose equivalent to TCE as vehicle through oral gavages once in a day for 5 consecutive days.

**Group II (negative control)**

Mice (*n* = 48) of this group were treated with 75 mg/kg b. w.t/day of TCE dissolved in double distilled water through oral gavage for 5 consecutive days once daily.

**Group III (irradiated control)**

Mice (*n* = 48) of this group were given double distilled water for 5 days (as in Group-I) and then exposed to 7.5 Gy dose of gamma radiation. This group served as irradiated positive control.

**Group IV (experimental)**

Mice (*n* = 48) of this group were given TCE (as in Group-II), and after 30 min of the last dose administration on day 5 such animals were exposed to 7.5 Gy gamma radiation.

### 2.6. Autopsy Schedule

Animals from all the above treated groups (I, II, III & IV) were regularly observed till 30 days for their weight change, any sign of sickness, morbidity, fur and skin changes, behavioral toxicity, any visible abnormalities, and mortality. All animals were necropsied at 12 hrs, 1, 3, 7, 15, and 30 days posttreatment for the evaluation of histological and biochemical variations in testes.

### 2.7. Histopathological Analysis

Testes were surgically removed at each autopsy interval from the necropsied animals of each group and weighed. One part of it was fixed in Bouin's fluid, and slides were prepared by routine procedure. Histological alterations in the testicular architecture were observed in the seminiferous tubules. For the measurement of weight index, average weight of both the testes from each animal was recorded. It was then correlated with the body weight of the animals. Tubular diameter of both peripheral and central seminiferous tubules was measured in the testes of each group at every interval with the help of an occular micrometer.

### 2.8. Biochemical Analysis

The remaining part of testis was used for biochemical analysis to measure lipid peroxidation (Ohkawa et al. [24] and glutathione (Moron et al. [25]) levels at each autopsy interval in all above groups.

### 2.9. Statistical Analysis

The results obtained in the present study were expressed as the mean ± SE. Statistical analysis (Student's *t*-test) was applied to find significant difference between the values of irradiated control and experimental.

## 3. Results

### 3.1. General

The mice exposed to 7.5 Gy gamma rays in the present study exhibited signs of radiation sickness within 2-3 days after radiation exposure. The irradiated animals showed reduction in food and water intake, ruffled hairs, diarrhea, watering of eyes, and irritability. About 85.72% mice died between 5 to 15 days post exposure in irradiated control group, and no animal could survive till the end of experiment. On the other hand, no symptoms of radiation sickness and mortality were observed in TCE pretreated irradiated group throughout the experiment.

### 3.2. Body Weight, Tissue Weight, and Testes Body Weight Ratio

Animals given DDW (Group I) and TCE alone (Group II) showed a continuous increase in body weight, tissue weight, and their ratio throughout the experiment. A considerable decline in all such parameters was observed in irradiated control animals (Group III) up to 15 day postirradiation. Similarly, TCE pretreated irradiated animals also showed progressive decrease up to day 7 of experiment in body weight, tissue weight, and weight index where values remained 71.91%, 61.43%, and 86.47% of initial, respectively; thereafter, these values increased till the end of experiment but without returning to normal (Table 1).

### 3.3. Tubular Diameter

After exposure to 7.5 Gy gamma rays, diameter of both central and peripheral seminiferous tubules in testes exhibited a significant and continuous decline from the initiation of treatment until day 15 posttreatment as compared to animals treated with DDW or TCE alone. Afterwards, a significant (*P* < .05) increase was recorded in the diameter of both the types of tubules, but the normal value could not be measured even till the end of experiment (i.e., day 30). A significantly higher tubular diameter was recorded at all the autopsy intervals in TCE pretreated group, but the pattern of change in tubular diameter was essentially similar to their respective control (Table 2).

### 3.4. Lipid Peroxidation

Lipid-peroxidation level in testes was found to be significantly (*P* < .001) higher in irradiated control animals (Group III) at all autopsy intervals as compared to vehicle-treated normal ones (group I). However, no animal could survive after irradiation (Group III) till day 17 posttreatment. LPO level also increased in TCE treated irradiated animals up to day 7 but the values were significantly (*P* < .05, *P* < .001) lower than their respective irradiated controls. Thereafter, a significant (*P* < .001) and progressive fall in LPO was observed on remaining intervals without the gaining normal level (Table 2).

### 3.5. Glutathione

No significant difference in the GSH content of testes was observed between normal, and TCE alone treated animals throughout the experiment. However, in irradiated control animals, a statistically significant decrease in GSH level was evident up to day 7 as compared to normal, but afterwards a significant (*P* < .001) increase in GSH was observed till day 15 posttreatment. TCE-treated irradiated animals also showed a similar mode of variation in GSH throughout the experiment, but the observed values were significantly higher at all autopsy intervals except at 12 and 24 hrs posttreatment (Table 2).

### 3.6. Testicular Histopathology

**Figure 1:**
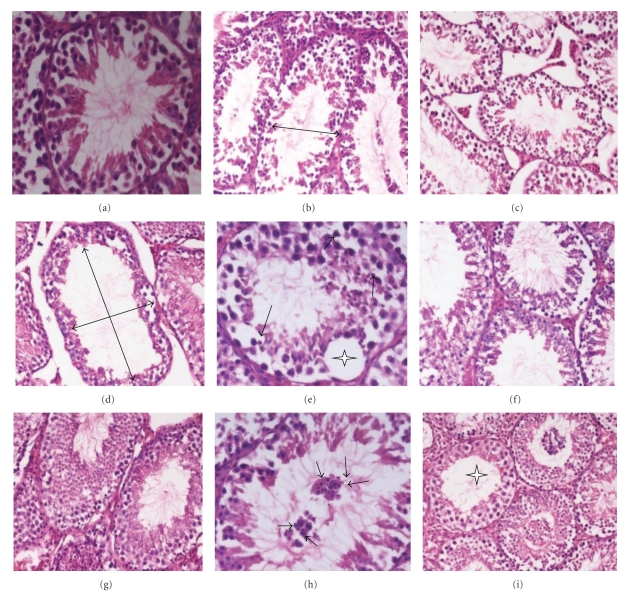
Modulation of radiation-induced histological changes in testes of Swiss albino mice by *T. cordifolia* extract. Photomicrograph (a) and (b) showing normal architecture of testis of sham irradiated and negative control, respectively. Photomicrographs (b, c, d, e) are showing radiation-induced pathological changes in irradiated control group from days 3, 7, 15, and 30, respectively, in the form of disrupted germinal epithelium, empty tubule (large arrow), intertubular oedema, pycnotic nuclei (small arrow), and intraepithelium vacuolation (asterick) with depleted germ cells population. Photomicrographs (f, g, h, and i) are showing better testicular architecture in TCE-treated irradiated group from days 3, 7, 15, 30, respectively, with a multinucleated formation of round spermatids (small arrow) at day 15 and almost normal structure at day 30 of experiment.

Histological sections of irradiated control mice showed drastic pathological lesions in tubular architecture when compared with mice treated with DDW or TCE alone. Distorted architecture of seminiferous tubules was seen in the form of shrunken tubules, exfoliation, intertubular oedema karyorrhexis, karyolysis, pycnotic nuclei, necrotic cells, and with degranulated cytoplasm in irradiated mice. These radiological symptoms were apparent from the initiation of treatment and the extent of damage intensified gradually up to day 7 posttreatment where cellular tires were completely distorted in most of the tubules along with decreased population of germ cells. Notably, TCE pretreatment rendered the quality as evident in the form of intact germinal epithelium, mild cytoplasmic vacuolization with the absence of karyolysis, pyknosis, and necrosis as well as increased germ cells number, and almost a normal testicular architecture was visualized by the end of experiment (Figure 1; photomicrograph (a–i)).

## 4. Discussion

Infertility has been a major medical and social preoccupation. The protective ability of the phytochemicals against radiation-induced male reproductive abnormalities may offer a new insight into the modification of testicular germ-cell radiosensitivity which may have implication in amelioration of testicular injuries. Therefore, the major concern of the present investigation is to assess the possible radioprotective capability of *Tinospora cordifolia* extract in clinical field against radiation-induced male reproductive dysfunctions. 

General observations in the present study clearly indicate that preirradiated treatment with TCE appreciably increased survival time of mice by 30 days without any symptoms of radiation-induced sickness. At the same time, irradiated animals exhibited sickness within 2–4 days with 71.43% mortality during 7 days and the remaining animals died within the next 10 days after exposure which might be occurred due to hematopoietic syndrome as suggested by others also [26, 27]. In view of the fact that the significant enhancement in the survival time may be owing to the protection afforded by TCE to the stem cell component of the bone marrow, which continued to supply the requisite number of cells in the survivors [28]. Body weight, testes weight, and testes-body weight ratio exhibited a similar mode of variation in both irradiated control and TCE-treated experimental animals. A notable recovery in body weight was ensued from day 7 onward in TCE-pretreated irradiated animals but without achieving the normal weight even till the end of experiment. These results suggest the possibility of protection of gastrointestinal system by TCE, since radiation-induced loss in body weight is due to the decrease in food and water intake due to gastrointestinal damage as also described by Griffiths et al. [29]. Some earlier studies from our laboratory also showed that administration of crude extracts of different herbal drugs reduced radiation-induced loss in body weight of animals [30–32], whereas decrease in testicular weight and testicular body-weight ratio after radiation exposure may be due to the actual loss in the germinal epithelial cells and not reflected by changes in the interstitial tissue or Sertoli cells. These findings may be further supported by our experiments in which spermatogenic cells in testes were found to be greatly reduced, while interstitial and Sertoli cells appeared almost unaffected by radiation exposure (unpublished data). Similar declining pattern in testicular weights and in weight index was also observed by Lin et al. [33] and Jagetia et al. [34] in lethally irradiated mice. 

Radiation is a potent toxicant and whole-body exposure of it can alter the general physiology of animal which might have an impulse over the normal histology and physiopathology of testes. Radiation can also downregulate its dual character, steroidogenic, and spermatogenic activity through the generation of oxidative stress, suppression of antioxidant mechanism, and by activating numerous molecular pathways involved in germ cells life and death decision making that ultimately altered normal testicular architecture [35]. In the present experiment, histological examination of seminiferous tubules of irradiated mice showed marked pathological alterations in the form shrinkage of tubules, distortion of cellular arrangement, exfoliation, severe intertubular oedema and hemorrhage in intertubular space, karyorrhexis, karyolysis, pycnosis, and necrosis. These findings are in close agreement with the earlier report of Pareek et al. [36] which documented the degenerative effects of gamma rays on spermatogenesis in lethally irradiated mice. The above pathological symptoms were manifested from the beginning of experimentation and increased progressively till day 15th in which germinal epithelium appeared flaccid and highly disorganized with total arrest of spermatogenesis. In addition to this, the nuclei, however, showed more destruction; they became shrunken and more concentrated with resultant karyorrhexis karyolysis and pycnosis. By the end of experiment, these nuclear and cytoplasmic changes led to necrosis and ultimately complete disappearance of the cells. Similar types of testicular injuries have also been reported by Zhang et al. [37], Koruji et al. [38], and Pande et al. [39] in lethally irradiated mice.

 Microscopic analysis of the testes in both irradiated control and TCE-treated animals revealed that peripheral as well as central tubular diameter of seminiferous tubules decreased progressively up to day 15 of irradiation and this might be caused by the spermatogenic cell loss and tubular disorganization. In TCE-treated irradiated animals, pathological lesions showed a similar pattern of alteration as in the irradiated control group, but their appearance was less prominent at all the autopsy intervals. TCE pretreatment rendered a high degree of recovery in spermatogenic cells, and almost a normal testicular architecture was reestablished by the end of experiment, but some pathological lesions like adhesion of tubules, mild cytoplasmic vacuolation, and less number of mature spermatozoa still persisted in the lumen of some tubules. A similar observation has also been reported while using *Panax ginseng* [40], *Podophyllum hexandrum *[41]* and Mentha *piperita [42] as radio-protector for the modulation of testicular injuries after irradiation.

The degenerative changes in testes observed at the early intervals of experiment in irradiated control may be due to immediate cell death and cellular death during their attempt to divide [43]. Intertubular oedema and hemorrhage in Intertubular spaces are caused due to direct effect of radiation on the testes, while other pathological alterations which were observed after 1 or 2 days may be due to the effect of radiation on the other parts of the body system [44]. Irradiated control animals showed shrunken and emptiness of seminiferous tubules that were associated with depletion in total germ cells population and breakdown of Sertoli cell-germ cell coordination links which might be responsible for the loosening and wavy appearance of tubular walls and tunica albuginea as suggested by others also [45, 46]. 

TCE preirradiation treatment was found to protect testicular cells very effectively which may be attributed to several factors such as efficient scavenging of free radicals, repair of DNA, membrane, and other damaged target molecules, and the replenishment of severely damaged or dead cells. The recruitment of cells to substitute the apoptatic and necrotic cells could also add to protection provided by TCE. In this study, the maximum protection is apparent after 3–7 days of radiation exposure which may be due to the fact that the optimal cellular concentration of free radical scavenging constituents of TCE is present in the system after 3–7 days.

 Understanding the protective mechanism accessible by medicinal plant extracts against the consequences of repeated oxidative stress in the male reproductive milieu is gaining wide attention in the present nuclear technological environment Agarwal and Said, 2005 [47]. Elevated levels of ROS may influence some transcription factors, enzyme activities, cell proliferation, and various important signal transduction pathways, leading to male reproductive dysfunctions (Kaur et al. [48]). 

Keeping this view, it pertinent to assess the possible anti-oxidative role of *T. cordifolia* extract against radiation-induced testicular oxidative stress. Radiation exposure induced a significant depletion in GSH levels at early intervals, which may be due to its enhanced utilization as an attempt to detoxify the acute radiation-induced free radical damage as glutathione is a major endocellular nonenzymatic antioxidant and executes its radioprotective function through free radical scavenging mechanism (Bump and Brown [49]. Depletion of GSH was lower in TCE pretreated animals as the animals of this group had a high level of phytoantioxidants after 5 days of TCE administration, therefore, less utilization of endogenous glutathione was observed. Afterwards, it tended to be utilized less due to the declining impact of radiation and endogenous reparative homeostatic activity. Previous findings from our laboratory [50, 51] strongly suggest that radiation-induced depletion of glutathione resulted in an enhanced LPO as also observed in testicular tissue by Faidan et al. [52]. Since biomembranes of testicular tissues are rich in polyunsaturated fatty acid content and radiation-induced damage is mediated by peroxidation of membrane lipids, therefore, the estimation of LPO is important to monitor oxidative damage to cellular membranes [53]. 

LPO produces a progressive loss of cellular integrity, fluidity of sperm membrane, and its motility, impairment in membrane transport function and disruption of cellular ion homeostasis in testes (Aitken and Baker, [54]). In the present study, both the irradiated control and experimental groups showed a gradual and continuous augmentation in the level of TBARS contents till day 15 postirradiation, which may be due to increased oxidative stress and decrease in body weight, organ weight, and protein value after radiation exposure as also suggested by Yadav et al. [55]. The perpetuation of cellular membrane integrity depends on protection or repair mechanism capable of neutralizing oxidative reactions. Preirradiation treatment of TCE significantly reduced LPO at all the autopsy intervals in comparison to irradiated control, which testifies to our belief that one of the possible mechanisms of radioprotection by TCE may be owing to the scavenging of free radicals generated by radiation exposure and prevents the formation of endoperoxidation. 

Our results suggest that TCE contains a very effective and natural antioxidant system (NAO) that is capable of preventing oxidative damage which is mediated principally through the generation of reactive oxygen species. The exact mechanism of action of TCE is not known. However, scavenging of free radicals and increased concentration of endogenous antioxidant system may be considered as important mechanisms of protection provided by TCE against radiation-induced damage to the testicular tissue. Tyagi et al. [56] imparts the support to this contention by the experiments on free radical scavenging, where TCE has been found to scavenge radiation-mediated OH and O_2_^−^ radicals. Earlier, Goel et al. [57] also reported the free-radical scavenging ability of aqueous extract of *T. Cordifolia*. The prophylactic action of TCE against radiation-induced reproductive and metabolic disorder may be due to the presence of several bioactive constituents like glycosides [58], phenolics [59], alkaloids [60], diterpenoid lactones [61], steroids [62], miscellaneous compounds [63], and so forth which may act through different mechanisms such as antioxidative [64], stimulation of cell proliferation [65], immunomodulation [66], and inhibition of lipid peroxidation [67]. 

Radioprotective efficacy may be due to combined/synergistic impact of these constituents rather than to a one single factor. Many studies around the world proved that the selection of a particular food plant, plant tissue, or herb for its potential health benefits appears to mirror its polyphenol and flavonoid composition. Polyphenols act as chain breakers or radical scavengers, which attribute to their antioxidant properties possibly through their O2^−.^ and singlet oxygen quenching ability as also noticed by Weiss and Landauer [68] and Hou et al. [69]. Most polyphenols, especially flavonoids are very effective scavengers of hydroxyl and peroxyl radicals [70]. The ability of flavonoids to scavenge free radicals and block lipid-peroxidation raises the possibility that TCE extract may act as protective factors against radiation mediated DNA damage. Correspondingly, a number of plant extracts have also been reported to react with free radicals and modulate free radical-mediated reactions mainly through their polyphenolic and flavonoid composition as reported by Prabhakar et al. [71] and Lia et al. [72].

Moreover, supplementation of TCE might have accountable for the increased concentration of phytoantioxidants which seem to be a responsible aspect for lowering the lipid-peroxidation because the basic cause of lipid-peroxidation is not only the free radicals but also the low levels of antioxidants that scavenge them. Alternatively, TCE might have increased the intracellular level of reduced glutathione, and stimulated the immune systems which could have provided protection against the radiation-induced mortality. Since significant radioprotection is obtained at a nontoxic dose of *T. cordifolia,* it may have an advantage over the contemporary radioprotector available at experimental level. Further, the studies are required to unravel the underlying mechanism of such plant against ROS-mediated damage for improving its efficiency better.

## 5. Conclusion

Based on the above promising results, it can be concluded that root extract of *Tinospora cordifolia* has the potential to mitigate the testicular injuries against lethal dose of gamma radiation, which in turn reflected in the form increased survival, inhibited pathological alterations, significant decline in LPO levels, and an enhancement in GSH content in TCE-pretreated irradiated group as compared to irradiated control group.

## Figures and Tables

**Table 1:** Variation in body weight, tissue weight, and testes-body weight ratio of mice after exposure to 7.5 Gy gamma radiation with (experimental) or without (irradiated control) TCE. The values represent mean ± S.E. The statistical significance was obtained between normal versus irradiated control and irradiated control versus experimental (*P*< * = .05, ** = .01, *** = .001).

Parameter	Group	Posttreatment autopsy intervals						
		0 hrs	12 hrs	24 hrs	3 days	7 days	15 days	30 days
Body weight (g)	I	25 ± 4.32	25 ± 5.21	26 ± 4.63	27.18 ± 5.67	28.62 ± 6.21	30.50 ± 6.73	32.78 ± 7.12
	II	24 ± 4.12	26.00 ± 4.21	28.65 ± 6.11	31.33 ± 6.23	33.66 ± 6.11	35.89 ± 5.81	38.66 ± 8.11
	III	24.08 ± 4.82	23.16 ± 5.02	21.39 ± 3.83	19.06 ± 1.51*	16.58 ± 4.47*	14.44 ± 5.7**	N.S.
	IV	24.53 ± 4.27	23.67 ± 4.92	22.69 ± 3.32	20.75 ± 2.9	17.63 ± 4.31	18.73 ± 5.31	21.17 ± 3.35*

Tissue weight (g)	I	0.159 ± 0.01	0.162 ± 0.05	0.166 ± 0.07	0.175 ± 0.08	0.185 ± 0.09	0.197 ± 0.06	0.213 ± 0.07
	II	0.154 ± 0.02	0.168 ± 0.06	0.187 ± 0.08	0.209 ± 0.09	0.226 ± 0.07	0.245 ± 0.11	0.267 ± 0.12
	III	0.154 ± 0.04	0.146 ± 0.021	0.134 ± 0.032	0.118 ± 0.021*	0.092 ± 0.001**	0.016 ± 0.002**	N.S.
	IV	0.153 ± 0.034	0.153 ± 0.033	0.143 ± 0.31	0.126 ± 0.019	0.094 ± 0.004	0.103 ± 0.011	0.119 ± 0.017

Weight Index	I	0.638 ± 0.05	0.639 ± 0.051	0.642 ± 0.048	0.644 ± 0.043	0.647 ± 0.053	0.649 ± 0.05	0.652 ± 0.07
	II	0.643 ± 0.04	0.649 ± 0.052	0.653 ± 0.047	0.662 ± 0.051	0.672 ± 0.048	0.684 ± 0.050	0.693 ± 0.052
	III	0.639 ± 0.051	0.630 ± 0.048	0.626 ± 0.046	0.619 ± 0.023	0.554 ± 0.053	0.734 ± 0.057	N. S.
	IV	0.623 ± 0.052	0.646 ± 0.048	0.630 ± 0.042	0.607 ± 0.049	0.533 ± 0.034	0.549 ± 0.037	0.562 ± 0.032

N.S.: Not survival, *Group *I: Normal/Sham-irradiated, *Group *II: Negative control, *Group *III: Irradiated Control, *Group *IV: Experimental.

**Table 2:** Variation in tubular diameter, lipid peroxidation, and glutathione in mice after exposure to 7.5 Gy gamma radiation with (experimental) or without (irradiated control) *Tinospoa cordifolia* extract. The values represent mean ± S.E. The statistical significance was obtained between normal versus irradiated control and irradiated control versus experimental (*P*< * = .05, ** = .01, *** = .001).

Parameter	Group	Posttreatment autopsy intervals					
		12 hrs	24 hrs	3 days	7 days	15 days	30 days
Peripheral tubular diameter (192 ± 16.2* *μ*m)	II	192 ± 16.52	192 ± 16.43	192.61 ± 16.44	194 ± 16.21	195.23 ± 33	196.11 ± 64
	III	147.24 ± 10.02*	143.13 ± 8.3*	135.2 ± 9.3*	120.48 ± 3.41**	98.12 ± 4.31***	N.S.
	IV	153.54 ± 10.11	150.02 ± 11.02	144.55 ± 9.71	134.44 ± 9.3	118.87 ± 6.2*	128.99 ± 4.52*

Central tubular diameter (157.74 ± 13.12* *μ*m)	II	167.37 ± 14.01	169.16 ± 13.161	170.63 ± 13.2	172.17 ± 14.22	173.47 ± 14.00	174.49 ± 13.22
	III	121.42 ± 4.47*	115.30 ± 6.11**	106.71 ± 7.12**	96.73 ± 3.27***	88.59 ± 4.12***	N.S.
	IV	133.63 ± 5.48	128.62 ± 4.32	121.8 ± 4.33	113.95 ± 5.61*	107.83 ± 6.18*	120.15 ± 7.58*

Lipid-per oxidation (1.63 ± 0.24* n mole/gm tissue)	I	1.52 ± 0.09	1.48 ± 0.11	1.36 ± 0.06	1.28 ± 0.13	1.17 ± 0.14	1.08 ± 0.22
	II	4.57 ± 0.73*	5.58 ± 0.78***	8.50 ± 0.72***	12.6 ± 0.83***	14.3 ± 0.93***	N.S.
	III	6.56 ± 0.86	3.59 ± 0.15*	3.99 ± 0.25***	5.29 ± 0.42***	5.07 ± 0.22***	5.03 ± 0.26***

Glutathione (5.862 ± 0.72∗ *μ* mole/gm)	II	5.884 ± 0.31	5.953 ± 0.28	6.032 ± 0.36	6.128 ± 0.41	6.168 ± 0.44	6.218 ± 0.39
	III	2.88 ± 0.52**	2.4 ± 0.43**	1.25 ± 0.18***	0.17 ± 0.03***	1.77 ± 0.13***	N.S.
	IV	3.669 ± 0.07	3.627 ± 0.52	2.954 ± 0.62*	2.089 ± 0.52*	3.248 ± 0.53*	4.121 ± 0.31***

NS: Non survival, *represents *Group *I: Normal/Sham-irradiated, *Group *II: Negative control, *Group *III: Irradiated Control, *Group *IV: Experimental.
